# Ultrasoft Long-Lasting Reusable Hydrogel-Based Sensor Patch for Biosignal Recording

**DOI:** 10.3390/bios14080405

**Published:** 2024-08-22

**Authors:** Alexandre Tessier, Shuyun Zhuo, Shideh Kabiri Ameri

**Affiliations:** 1 Department of Electrical and Computer Engineering, Queen’s University, Kingston, ON K7L 3N6, Canada; 2 Centre for Neuroscience Studies, Queen’s University, Kingston, ON K7L 3N6, Canada

**Keywords:** ultrasoft hydrogel, reusable sensor, electrophysiological recording, sensor-skin interface, signal-to-noise ratio

## Abstract

Here, we report an ultrasoft extra long-lasting, reusable hydrogel-based sensor that enables high-quality electrophysiological recording with low-motion artifacts. The developed sensor can be used and stored in an ambient environment for months before being reused. The developed sensor is made of a self-adhesive electrical-conductivity-enhanced ultrasoft hydrogel mounted in an Ecoflex-based frame. The hydrogel’s conductivity was enhanced by incorporating polypyrrole (PPy), resulting in a conductivity of 0.25 S m^−1^. Young’s modulus of the sensor is only 12.9 kPa, and it is stretchable up to 190%. The sensor was successfully used for electrocardiography (ECG) and electromyography (EMG). Our results indicate that using the developed hydrogel-based sensor, the signal-to-noise ratio of recorded electrophysiological signals was improved in comparison to that when medical-grade silver/silver chloride (Ag/AgCl) wet gel electrodes were used (33.55 dB in comparison to 22.16 dB). Due to the ultra-softness, high stretchability, and self-adhesion of the developed sensor, it can conform to the skin and, therefore, shows low susceptibility to motion. In addition, the sensor shows no sign of irritation or allergic reaction, which usually occurs after long-term wearing of medical-grade Ag/AgCl wet gel electrodes on the skin. Further, the sensor is fabricated using a low-cost and scalable fabrication process.

## 1. Introduction

As demands for the advancement of medical and personal health care, human–machine interfaces (HMI), and Internet of Things (IoT) technologies grow, and novel sensors are being developed, the all-in-one integration of characteristics such as reliability, low cost, and user-friendliness has remained challenging. In recent years, various types of wearable sensors have been developed, with one of their main sensing capabilities being electrophysiological recording, including electromyography (EMG), electrocardiography (ECG), electroencephalography (EEG), and electrooculography (EOG) [1,2,3,4,5,6,7,8,9,10,11]. The mechanical characteristics of these sensors not only play a role in making sensors comfortable to wear but, more importantly, significantly influence the performance of the sensor by affecting motion artifacts, impacting the sensor-skin interface impedance and, consequently, signal-to-noise ratio (SNR). In the manufacturing of these wearable sensors, different materials such as metal, semiconductor particles and films, conductive polymers, and composites have been utilized [12,13,14,15,16,17,18]. Among these materials, conductive polymer composites have shown promise for commercialization due to the low cost and scalability of the manufacturing process, tunable mechanical and electrical properties, biocompatibility, reusability, and robustness of wearable sensors made of conductive polymer composites. However, most conductive polymers and composites are mechanically stiff in comparison with human skin. For example, most of the reported conductive polymers and composites that are widely used for biophysiological sensing, such as poly(3,4-ethylenedioxythiophene): polystyrene sulfonate (PEDOT:PSS) and polypyrrole (PPy), are intrinsically stiff which limits the conformability of the fabricated sensor to the skin. At the same time, hydrogel composites composed of different types of polymer matrixes and conductive fillers like carbon nanotubes, carbon black, silver nanoparticles, graphene flakes, and reduced graphene oxide [19,20] can be made extremely soft due to their high water content.

Hydrogels with characteristics such as outstanding softness and stretchability, biocompatibility, tunable conductivity, and simple synthesis have attracted interest for application in wearable sensors. Hydrogels contain high amounts of water and are, therefore, ionically conductive [21,22]. In addition, they can be self-adhesive, which helps in forming stable contact at their interface with the skin. The high conductivity and softness of the hydrogel-based sensors assist in achieving lower skin-sensor interface impedance and, consequently, better signal quality and larger SNR [19,20,23]. Further, unlike commercial and medical-grade wet gel silver/silver chloride (Ag/AgCl) electrodes and many other commercial wearable sensors, which cause irritation and allergic reactions because of the use of aggressive adhesives to hold them in place, hydrogels are self-adhesive and usually do not cause irritation [24,25]. Hydrogels are also generally biocompatible and have been used for wound dressing, drug delivery, and tissue engineering [5,26,27]. To improve the conductivity of hydrogels, various salts, intrinsically conductive polymers, metal particles/wires, and carbon nanomaterials have been incorporated into hydrogel networks [28,29,30]. However, adding mechanically stiff fillers to ultrasoft hydrogels results in a significant increase in the overall stiffness of the hydrogel. Further, since the hydrogel contains considerable amounts of water, it dries out quickly, which results in a drop in the electrical conductivity of the hydrogel and also degrades its mechanical properties.

Various methods are used to address this issue, such as adding water retention agents like salts and glycerol and encapsulation of hydrogels [29,31,32,33]. Despite the improvements made in fabricating hydrogel sensors for electrophysiological measurements, developing cost-efficient, long-lasting, reusable, and robust hydrogel sensors with high electrical conductivity, softness, and mechanical and electrical stability requires further studies and advancements. 

Here, we report the development of a soft, highly conductive, long-lasting, reusable, low-cost, self-adhesive hydrogel-based sensor for long-term electromyography and electrocardiography recording with high-quality and low-motion artifacts. Compared with the previously reported hydrogel-based sensors, our developed hydrogel sensor shows its benefits through (1) a simple and efficient fabrication process, (2) a long lifetime of more than 2 months and reusability, and (3) the direct function of hydrogel for electrophysiological sensing rather than only serving as a soft substrate or interface layer between the soft electronic layers. The combination of the aforementioned characteristics with the ultra-softness and self-adhesion of our developed hydrogel sensor ensures a stable interface of the sensor to the skin and enables high-quality signal recording with low-motion artifacts, suitable for long-term ECG recording. The sensor shows a very good quality of signal recording, high SNR, low-motion artifact, and long lifetime. The sensor can be stored for over 2 months and be reused later after rehydrating it. The method of fabrication of this sensor is simple, time-, and cost-effective. Due to the softness of the hydrogel-based sensor, it forms conformal contact with the skin and, as a result, allows achieving low sensor-skin interface impedance, high quality of signal recording with higher SNR than gold-standard medical-grade Ag/AgCl wet gel electrodes, and minimized motion artifacts. 

## 2. Materials and Methods

Materials: Gelatin (from porcine skin, gel strength ~300 g Bloom, Type A), pyrrole (98%), potassium persulfate (KPS, ≥99.0%), poly (ethylene glycol) diacrylate (average Mn 700), and 2,2-Diethoxy acetophenone were purchased from Sigma Aldrich. Acrylamide (98+%) was purchased from Thermo Scientific. Ecoflex and adhesive polydimethylsiloxane, PDMS (LiveoTM MG7-9850) were purchased from TAP Plastics and the DOW chemical company, respectively.

Methods: Fabrication of hydrogels: The hydrogel precursor was made by mixing 10 wt% gelatin, 10 wt% acrylamide, 0.8 wt% poly (ethylene glycol) diacrylate as a crosslinker, 0.02 wt% 2,2-Diethoxy acetophenone as an ultraviolet (UV) initiator, and approximately 79.2 wt% deionized (DI) water at 50 °C for 15 min to ensure homogenous consistency of all the components. Then, the hydrogel precursor was added to the PDMS sensor patch to fabricate sensors or into molds for mechanical, electrical, and adhesion measurements, followed by photopolymerizing under 365 nm UV light (ANYCUBIC wash and cure station, Shenzhen, China, 25 W) for 1 h. The resultant hydrogel was then soaked in 8 wt% pyrrole solution for 1 h and in 5 wt% potassium persulfate solution for 30 min and dipped in DI water three times for 15 min each to remove unreacted chemicals. Hydrogels with different PPy contents were fabricated using 2–8 wt% pyrrole aqueous solution.

Fabrication of hydrogel-based sensors: A mold was first designed with the reference and working electrodes 2 cm apart (the surface area of each electrode was set to be 0.79 cm^2^) and was then printed using a three-dimensional (3D) printer (Statasys printer, model F190CR, Stratasys Inc., Eden Prairie, MN, USA) with acrylonitrile butadiene styrene (ABS) material. Ecoflex precursor was prepared by mixing two Ecoflex agents at a ratio of 1:1 followed by de-bubbling using a vacuum pump (1 stage vacuum pump, RS-1), which was poured in the printed mold and heated at 50 °C for 30 min on a hotplate (Cole-Parmer, RK-04671-26, Montréal, QC, Canada). After curing, the Ecoflex patch was de-molded from the mold and covered with a thin layer of adhesive PDMS precursor (with a ratio of 1:1 of two agents) followed by heating at 50 °C for 20 min. Then, the hydrogel was made within the cavity of the patch to form the working and reference electrodes.

## 3. Results and Discussion 

**Figure 1:**
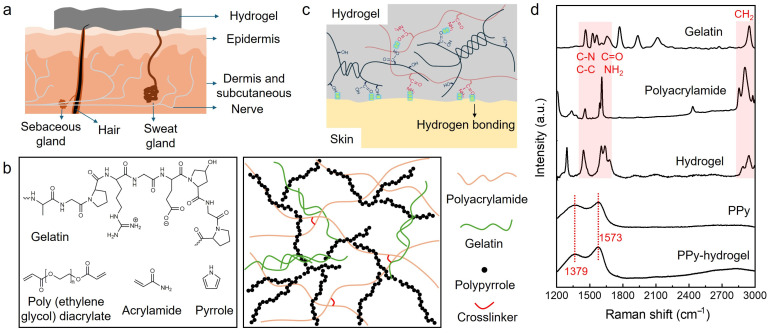
(**a**) The schematic of a conformal hydrogel sensor at the interface with the skin. (**b**) The chemical structure of the components of the hydrogels and the illustration of the hydrogel networks. (**c**) Illustration showing the self-adhesion property of the hydrogels due to hydrogen bonding interactions within the hydrogel network and with substrates. (**d**) Raman spectra of gelatin, polyacrylamide, hydrogel containing gelatin and polyacrylamide, PPy, and PPy-hydrogel.

Hydrogels have been widely used in wearable sensors and electronic skins. Figure 1a is the schematic of a hydrogel sensor with conformal contact to the skin, which results in reducing the sensor-skin interface impedance and consequently improving the signal-to-noise ratio. These conformable sensors on the skin require hydrogels to be soft and robust to withstand various deformations. The hydrogels developed in our work are crosslinked both physically and chemically. As shown in Figure 1b, the hydrogel is prepared by photoinitiated free radical addition of acrylamide, which is further chemically crosslinked by poly (ethylene glycol) diacrylate, while the gelatin is self-crosslinked by forming triple helix structures through interchain hydrogen bonds, resulting in the 3D hydrogel networks. The high water content of 79.2 wt% and moderate crosslinking contribute to the softness and integration of the hydrogels. 

In addition to the sensor-skin interface, the conductivity of the sensor plays an important role in the sensing performance of wearable devices, where high conductivity can enhance the signal quality. To achieve high conductivity, a biocompatible conductive polymer, polypyrrole (PPy), was introduced into the hydrogel networks. Figure 1b shows a schematic of the crosslinked hydrogel containing PPy, where the connected PPy forms a conducting path for the electrons. In addition, reversible hydrogen bonding interactions formed between the hydroxyl, amino, and carbonyl groups of gelatin and polyacrylamide, which contributed to the self-adhesive property of the hydrogel (Figure 1c). Raman spectroscopy was used to investigate the chemical structure of hybrid hydrogels. As shown in Figure 1d, gelatin and polyacrylamide show peaks at various wavenumbers from 1400 to 1650 cm^−1^, which can be ascribed to C-C and C-N stretching vibrations (1604 cm^−1^, 1453 cm^−1^) and C=O stretching and N–H bending vibrations (1650 cm^−1^) [34,35,36]. The characteristic CH_2_ stretching vibration peak appeared at 2905–2942 cm^−1^ [37]. These peaks were also observed in hydrogels composed of gelatin and polyacrylamide, indicating the integration of these two components in the hydrogel networks. After incorporating PPy into the hydrogel, the typical intense tangential resonance absorption band (G band) at 1573 cm^−1^ and a defect band (D band) at 1379 cm^−1^ were found, which represent the C=C symmetry stretching and inter-ring (C–C) stretching of PPy, respectively [38,39].

**Figure 2:**
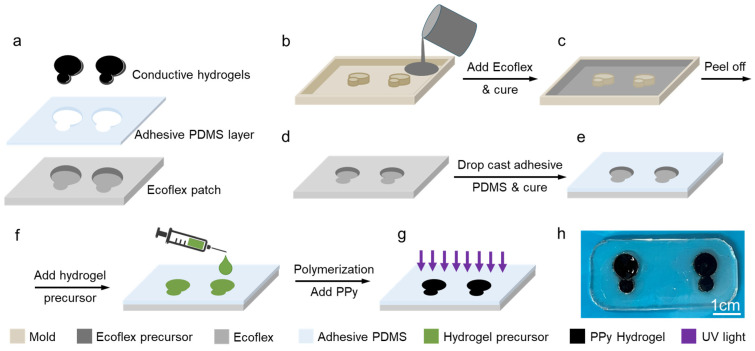
Hydrogel-based sensor fabrication process. (**a**) The integration of silicone-based cast, hydrogel, and adhesive PDMS to fabricate the sensor. (**b**) A mold was printed using a 3D printer; (**c**) Ecoflex precursor was poured into the mold and cured; (**d**) then the Ecoflex sensor frame was de-molded; and (**e**) was covered with a layer of adhesive PDMS and cured. (**f**) Hydrogel precursor was added to the wells in the sensor’s frame, and (**g**) was polymerized by UV light and finally incorporated with PPy. (**h**) The photograph of the fabricated hydrogel sensor scale bar is 1 cm.

The developed sensor is composed of a soft Ecoflex frame, a thin layer of adhesive polydimethylsiloxane (PDMS), and conductive hydrogels, as shown in Figure 2a. The adhesive PDMS was cast on the surface of the matrix and used to ensure a stable sensor-skin interface. The sensor was fabricated through a low-cost, simple, and scalable process of molding and casting. The hydrogel precursor was poured into the designated spaces in a fabricated Ecoflex frame, followed by its polymerization and incorporation of PPy. To perform ECG and EMG, at least three sensors/electrodes are required: reference, working, and ground electrodes, from which the reference and working electrodes must be at least 2 cm away, and the ground electrode must be placed somewhere on the body that is physically distant from the origin of the signal of interest. To fabricate hydrogel-based sensors, a frame composed of Ecoflex with the reference and working electrodes embedded in it was fabricated. The total surface area of each sensor node was made to be 0.79 cm^2,^ which is only 45% of the commercial medical-grade Ag/AgCl wet gel electrode (1.77 cm^2^). The ground electrode was a separate stand-alone electrode. Figure 2 shows the process of the fabrication of the sensor. Ecoflex precursor was poured into the printed mold to form a soft and stretchable frame after curing (Figure 2b,c), hosting the working and reference electrodes. After demolding, the Ecoflex surface was coated with a thin layer of adhesive PDMS to allow self-adhesion of the sensor patch to the skin (Figure 2d,e). Then, a conductive hydrogel containing PPy was fabricated in the cavities of the Ecoflex frame to form hydrogel sensors (Figure 2f,g). The optical image in Figure 2h shows the fabricated sensor with transparent and colorless patches and black hydrogel electrodes. 

**Figure 3:**
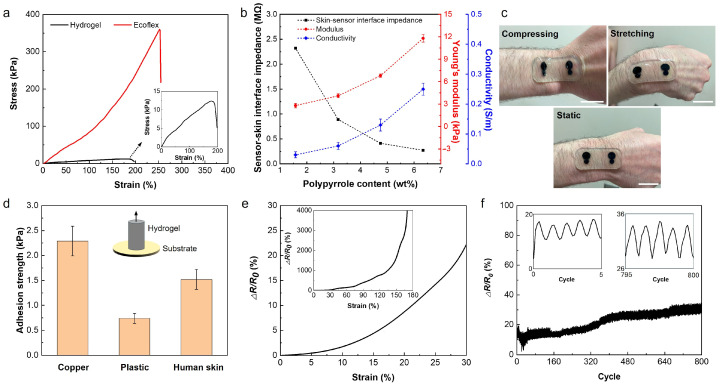
Mechanical and electrical properties of the hydrogel. (**a**) Stress-strain plot of hydrogel and Ecoflex. (**b**) Sensor-skin interface impedance at 20 Hz, Young’s modulus, and conductivity of hydrogels prepared by soaking in solutions with different pyrrole contents. (**c**) Photographs showing hydrogel-based sensors adhered to human skin in a static state and under strain and compression. Scale bar is 2 cm. (**d**) The measured adhesion strength of 6-PPy/hydrogel to different materials. (**e**) Changes in the electrical resistance of the 6-PPy/hydrogel by applying tensile strain. (**f**) Changes in the electrical resistance of the 6-PPy/hydrogel during the cyclic strain test at 30% of applied tensile strain for 800 cycles.

In order to study the mechanical characteristics of the sensor, the strain-stress behavior of both Ecoflex and hydrogel was studied using a Univert CellScale tensile machine with 50 N and 10 N load cells, respectively. The strain-stress curves of the sensor patch and hydrogel are shown in Figure 3a. The plots indicate the hyper-elasticity of the sensor patch and the ultra-softness and high stretchability of the hydrogel. Young’s moduli of the Ecoflex and hydrogel were measured to be 89.1 kPa and 12.9 kPa, and the elongation to break was 255% and 190% for the patch and the hydrogel electrodes, respectively. Compared with human skin, which has a modulus of 60–850 kPa and elongation within 30% tensile strain, our developed hydrogel-based sensor with a lower modulus and higher stretchability demonstrates high potential for forming stable and conformable interfaces with the skin for wearable applications [40,41]. Figure S1 shows the deformability of the sensor placed on the wrist of the subject with strain applied by flexing the wrist. Because of the mechanical softness and self-adhesion of the hydrogel-based sensor, its lamination on different parts of the body with different topologies, including the chest, arm, forearm, leg, ankle, and wrist, is feasible, and this is shown in Figure S2. These results indicate that the hydrogel-based sensor forms a stable and robust interface with the skin, which could decrease the susceptibility of the sensors to motion. 

The sensing quality of the wearable electrophysiological sensors is mainly impacted by the electrical conductivity of the sensor and conformability of the sensor to the skin, which both affect the sensor-skin interface impedance and, consequently, SNR. Despite the hydrogel’s limited ionic conductivity due to the low concentration of the ions, further improvement in the conductivity of such hydrogels is required for high performance in electrophysiological signal recordings. To increase the conductivity of the hydrogel, we used PPy as a conductive polymer within the hydrogel network. Adding PPy increases the conductivity of the hydrogel but also increases its stiffness. Therefore, there is a tradeoff between increasing the conductivity of the hydrogel by adding PPy and maintaining the mechanical softness and conformability of the sensor to the skin. Figure 3b shows the conductivity of the hydrogels versus the polypyrrole content. The PPy contents within the hydrogel range from 1.58 wt% to 6.34 wt%, resulting from soaking the hydrogel in pyrrole aqueous solutions containing 2–8 wt% of pyrrole. As it can be observed, the conductivity of the hydrogel increases by increasing the PPy content from 0.03 ± 0.01 S m^−1^ at 1.58 wt% of PPy to 0.25 ± 0.02 S m^−1^ at 6.34 wt% of PPy. It should be noted that no tests were performed beyond 6.34 wt% due to the limited solubility of the pyrrole in DI water, as seen in Figure S3. Meanwhile, Young’s modulus of hydrogel increases from 3 kPa to 12.9 kPa, and sensor-skin interface impedance decreases from 2.32 MΩ to 140 KΩ when the PPy content increases from 1.58 wt% to 6.34 wt% (Figure 3b and Figure S4). The sensor-skin interface impedance was measured by placing the hydrogel-based sensor on the forearm. The elongation to break off the hydrogel was also affected by the PPy content, which decreased from 500% to 180% by increasing PPy contents from 1.58 wt% to 6.34 wt%, as can be seen in Figure S5. 

Based on the obtained results, the hydrogel with 6.34 wt% of PPy (6-PPy/hydrogel) was used for the fabrication of the sensors. Hydrogels have self-adhesive properties that help form a more stable interface with the skin and minimize motion artifacts during mobile sensing. Figure 3c demonstrates the effective adhesion of the hydrogel sensor to the skin, where no delamination is observed when the hydrogel sensor goes through various deformations like stretching and compressing. Such a robust sensor-skin interface can be ascribed to the self-adhesion property of the hydrogel and the adhesive PDMS with a reported adhesion force of 1.1 N/cm^2^ [42]. Our results show that the 6-PPy/hydrogel can adhere to different materials, including plastics, metal, and human skin. Figure 3d shows the measured adhesive strength of 6-PPy/hydrogel on different surfaces such as metal, plastic, and skin. The adhesion strength between 6-PPy/hydrogel and copper is higher than that between the hydrogel and plastic and the skin. The adhesion strength between the skin and 6-PPy/hydrogel is 1.52 ± 0.2 kPa, which is sufficient for the adhering sensor to the skin stably. The self-adhesion of the hydrogel can be ascribed to the dynamic hydrogen bonding interactions between the hydrogel and the substrates.

The change in the electrical conductivity of 6-PPy/hydrogel due to the applied tensile strain was measured and is presented in Figure 3e. The 6-PPy/hydrogel shows a 22.5% increase in the electrical resistance at an applied strain of 30%. The electrical stability of the sensors is important for long-term measurements, which is affected by the anti-fatigue property of the used electrode materials. To study the fatigue behavior of the 6-PPy/hydrogel, a strain cycling test was performed (Figure 3f). The changes in the electrical resistance of the 6-PPy/hydrogel for 800 cycles of 30% tensile strain suggest that the resistance change of the hydrogel is stable. A resistance drift of less than 40% was observed before reaching a plateau after 400 cycles, which could be ascribed to the gradual drying out of the high water content hydrogels. We addressed this issue by casting a 6-PPy/hydrogel in Ecoflex to facilitate water retention and increase the lifetime of the sensor. 

**Figure 4:**
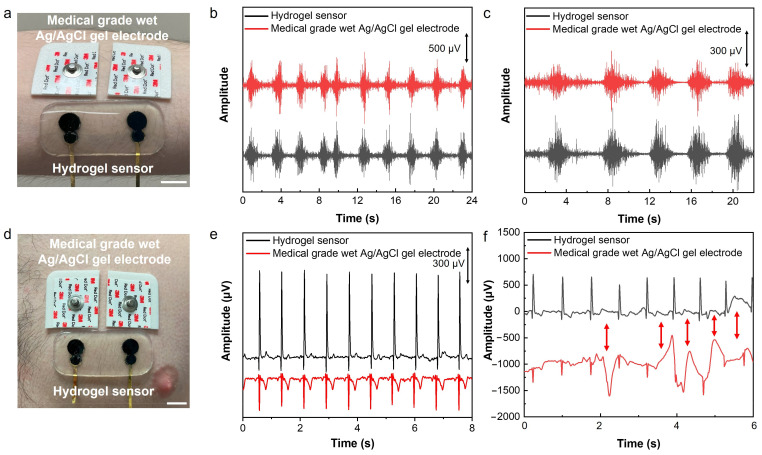
EMG and ECG recording using the developed hydrogel-based sensor. (**a**) The placement of the sensor and medical-grade wet Ag/AgCl gel electrodes on the forearm for EMG recording. (**b**) Recorded EMG signals using a hydrogel sensor and medical-grade wet Ag/AgCl gel electrodes. (**c**) EMG signals recorded after exercise and sweating on the skin. (**d**) The placement of the hydrogel sensor and medical-grade wet Ag/AgCl gel electrodes on the chest for ECG recording. (**e**) Recorded ECG signals using a hydrogel sensor and medical-grade wet Ag/AgCl gel electrodes concurrently. (**f**) ECG signals recorded during motion induced by poking the chest with a glass rod in the vicinity of the sensor. The scale bar is 1 cm.

The developed sensor was used for electrophysiological signal recording. Electromyography (EMG) and electrocardiography (ECG) were performed by placing the sensor on the forearm flexor muscle and chest, respectively. As can be observed in Figure 4, in both cases, a set of medical-grade silver/silver chloride (Ag/AgCl) wet gel electrodes was also placed next to our developed sensor for concurrent signal recording. EMG signals were recorded while the subject was squeezing a hand grip. The result shows a clear EMG signal with an SNR of 26.92 dB recorded by our developed hydrogel sensor in comparison to 22.97 dB recorded by the medical-grade electrodes (Figure 4b).

To study the performance of the sensor for EMG sensing during daily activities like physical exercise, sensor-skin interface impedance was measured before and after exercise and sweating. As shown in Figure S6, there is no significant difference between the sensor-skin interface impedances tested before exercise and after running for 20 min, indicating the robustness and high stability of the hydrogel-based sensor-skin interface. This high-quality interface and low sensor-skin impedance contribute to the high performance of the hydrogel sensor in EMG recording, as shown in Figure 4c.

The application of the developed sensor for performing ECG was also demonstrated. The hydrogel sensor was placed on the chest next to a pair of medical-grade wet gel Ag/AgCl electrodes as the gold standard for recording ECG signals simultaneously (Figure 4d). The signals with essential ECG characteristic peaks of P, Q, R, S, and T were recorded using both sets of sensors and electrodes; the results in Figure 4e demonstrate significantly higher signal amplitude measured by the hydrogel sensor than the medical-grade electrodes. As a result, the SNR of the ECG signals recorded using our sensor was 11.39 dB higher than that measured by the medical-grade wet electrodes (33.55 dB compared to 22.16 dB). A motion artifact study was performed while recording the ECG concurrently using our sensor and medical-grade wet gel electrodes. The motions were induced by poking the skin using a glass rod in the vicinity of the sensor and medical-grade electrodes. The results presented in Figure 4f show that our sensor is significantly less susceptible to motion, while the medical-grade electrodes have shown significant artifacts in the ECG signals. The lower motion artifact of the hydrogel-based sensor is due to its high deformability and softness, as well as its efficient self-adhesion and conformability to the skin’s microscopic features, which ensures a stable sensor-skin interface and high sensing performance. 

**Figure 5:**
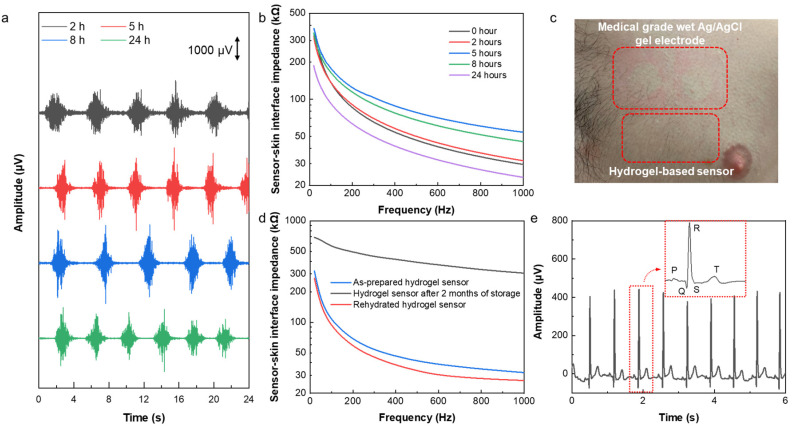
Long-term sensing capability and reusability of the hydrogel sensor. (**a**) EMG signal recording and (**b**) sensor-skin interface impedance after 2, 5, 8, and 24 h of wearing the hydrogel-based sensor. (**c**) Photograph showing that there was no skin irritation due to using our sensor, while skin irritation was caused by medical-grade Ag/AgCl wet gel electrodes. (**d**) Sensor-skin interface impedance of the as-prepared hydrogel sensor, a used sensor after storage for 2 months in an ambient environment, and the rehydrated used and stored sensor within the frequency range of 20–1000 Hz. (**e**) ECG signals recorded by the reused and rehydrated sensor. The inset shows the ECG characteristic peaks.

Wearable sensors have been successfully applied for long-term health monitoring during daily life. Signal quality, comfort, and skin reactions are the most important aspects of long-term electrophysiological sensing. To study the capability of the hydrogel-based sensor for long-term monitoring, it was worn for 24 h, and EMG was recorded after wearing it on the forearm for 2, 5, 8, and 24 h. As can be seen in Figure 5a, the signal quality of the recorded EMG signals did not degrade over time but even slightly improved. This is because of the self-adhesion and enhanced conductivity of the hydrogel sensors, as well as the high stability of the sensor-skin interface due to the existence of the Ecoflex frame that acts as an encapsulation. Thus, the sensor demonstrated its capability of being used for long-term sensing. Figure 5b shows the changes in the sensor-skin interface impedance in the frequency range of 20 Hz to 1 kHz by time. This result shows that the interface impedance decreases over time in the first 24 h after wearing the sensor, which can be attributed to sweating and increasing the water and salt content at the interface of the sensor with the skin. After removing the sensor, as shown in Figure 5c, there was no sign of irritation and/or allergic reaction to the sensor. However, in the case of medical-grade Ag/AgCl wet gel electrodes (3M), a clear indication of skin irritation was observed. 

Finally, the long-lasting property and extended lifetime of the sensor were investigated. In order to study the reusability and lifetime of the hydrogel sensor, the sensor-skin interface impedance and ECG signals were measured after storing a used sensor for a period of 2 months in an ambient environment. The hydrogel sensors inevitably dry out even when stored in a sealed container, which limits the lifetime and reusability of the hydrogel-based sensors. However, the developed sensor can be used and stored for an extended period of time and can restore its full electrical performance after the rehydration process. As shown in Figure S7, a used sensor that was stored for 2 months was fully rehydrated in 15 min by adding a drop of water on each electrode in the sensor to rehydrate it. Figure 5d shows the sensor-skin interface impedance of the rehydrated hydrogel sensor, which decreased significantly from 304.9 kΩ before rehydration to 26.7 kΩ after rehydration at 1 kHz, and it is very similar to that of the as-prepared hydrogel sensor, indicating a full restoration of the used and stored sensors. As a result, the ECG signals recorded by the reused and rehydrated sensor demonstrated high amplitude and clear characteristic P, Q, R, S, and T peaks (Figure 5e). These results demonstrate the extended lifetime and high reusability of the hydrogel sensor, making it a good candidate for long-term health monitoring.

## 4. Conclusions

To conclude, we demonstrated the fabrication of a reusable, long-lasting, conformal, low motion artifact hydrogel sensor. The fabrication of this sensor, which is based on casting and molding, is simple and low-cost. The Ecoflex frame, which acts as an encapsulation for the hydrogel, significantly improves the water retention capability of the hydrogel and its capability for long-term recording during daily activities in different environments with different humidity levels and temperatures. The developed sensors are reusable and have an extended lifetime. The used sensors can be stored for 2 months in the ambient environment and at room temperature and can be used again after rehydrating them without any degradation in their performance in electrophysiological signal recording. The application of the sensor to ECG and EMG was demonstrated. The sensors have shown significantly greater SNR and lower motion artifacts than gold-standard medical-grade electrodes. In addition, no skin irritation was observed when the hydrogel-based sensor was placed on the skin for a long time. The sensor has potential applications in medical and health care, as well as in human–machine interfaces. 

## Data Availability

The original contributions presented in the study are included in the article/Supplementary Material, further inquiries can be directed to the corresponding author/s.

## Supplementary Materials

The following supporting information can be downloaded at: https://www.mdpi.com/article/10.3390/bios14080405/s1, Figure S1: Hydrogel-based sensor adhered to human skin; Figure S2: Hydrogel-based sensors placed on various parts of the body; Figure S3: Photos showing the pyrrole aqueous solutions with different pyrrole contents; Figure S4: Sensor-skin interface impedance of the hydrogel-based sensors; Figure S5: Strain-stress curves of hydrogels with different polypyrrole contents; Figure S6: Sensor-skin interface impedance of hydrogel-based sensors before and after exercise; Figure S7: The photos showing a sensor after being stored and rehydrated.
