# Supplementary material for: Ultrasoft Long-Lasting Reusable Hydrogel-Based Sensor Patch for Biosignal Recording
Source: Biosensors (Basel). 2024 Aug 22;14(8):405. doi: 10.3390/bios14080405 (PMC11352425; doi:10.3390/bios14080405)
Supplement: Supplementary file 1 [file biosensors-14-00405-s001.zip › biosensors-3125340-supplementary.pdf]

biosensors

MDPI

Supporting Information

Ultrasoft Long-Lasting Reusable Hydrogel-Based Sensor Patch for Biosignal Recording

Alexandre Tessier [1,†], Shuyun Zhuo [1,†] and Shideh Kabiri Ameri [1,2,*]

[1] Department of Electrical and Computer Engineering, Queen's University, Kingston, ON K7L 3N6, Canada
[2] Centre for Neuroscience Studies, Queen's University, Kingston, ON K7L 3N6, Canada
* Correspondence: shideh.ameri@queensu.ca
† These authors contributed equally to this work.

Characterizations:

Mechanical tests: Tensile tests were conducted on both hydrogel samples and the Ecoflex, using a Univert CellScale tensile machine with 50 N and 10 N load cells. The hydrogel and Ecoflex samples (3 cm * 1 cm * 1 mm) were stretched at a strain rate of 10 mm/min till break. The modulus was calculated from the linear slope of the stress-strain curves within 30%.

Adhesion test: Cylinder-shaped hydrogel samples with a diameter of 5 mm were adhered ondifferent surfaces such as copper, plastic, and human skin and were stretched perpendicularly to detach from the surface. The Force was recorded by the Univert CellScale tensile machine.

Electrical test: The resistance of the hydrogels with 1.58-6.34 wt% PPy was measured using a digital multimeter (Fluke, 8050A). Conductivity was calculated from the resistance of the hydrogel sample. The resistance changes of the hydrogel with strain were recorded by the digital multimeter while stretching the sample to break. Cyclic resistance changes were also tested during loading-unloading cycles with a tensile strain of 30% for 800 cycles.

Raman test: Gelatin, polyacrylamide, hydrogel samples composed of gelatin and polyacrylamide, PPy, and PPy-hydrogel were measured using a Renishaw inVia Raman microscope scanning from 1200 cm^{-1} to 3000 cm^{-1}. Polyacrylamide was synthesized by the photopolymerization of 10 wt% acrylamide in DI water. A hydrogel sample was used without adding PPy. PPy was obtained by dispersing pyrrole in DI and adding potassium persulfate for polymerization, followed by washing with DI water to remove unreacted chemicals and drying at 60 °C. All the samples used for the Raman test were dried at 60 °C on a hotplate.

Sensor-skin interface impedance: The sensor-skin interface impedance was measured by placing the sensor on the forearm, and the impedance was recorded by an LCR meter (Keysight, E4980AL) within the frequency range of 20-1000 Hz. No skin preparation was performed.

EMG and ECG signal recording: The hydrogel sensor was attached to the chest for ECG measurement and to the forearm flexor muscle for EMG measurement. A ground hydrogel electrode was used and placed on the wrist and on the elbow for ECG and EMG, respectively. The electrodes were connected to an OpenBCI board, and the data were wirelessly transferred to a computer. A pair of medical grade wet gel Ag/AgCl electrodes (3M) was used as the gold standard and attached next to our hydrogel-based sensor for recording ECG/EMG signals simultaneously.

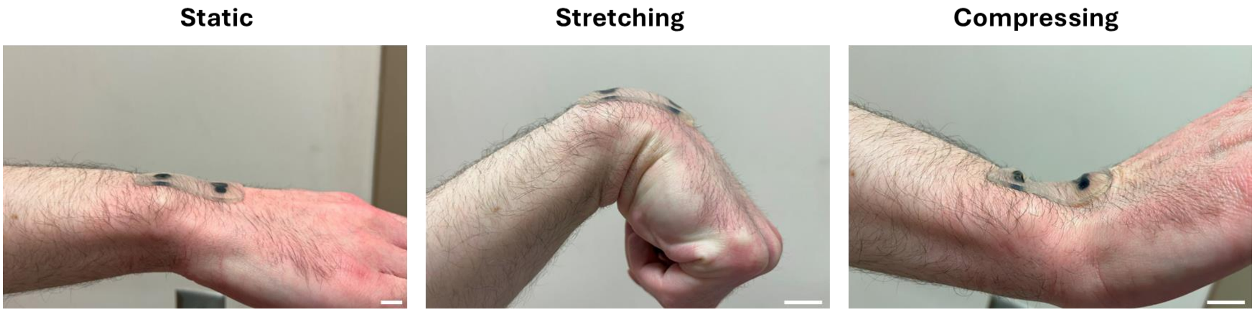

Figure S1. Hydrogel-based sensor adhered to human skin in a static state and under strain and compression. Scale bar, 1 cm.

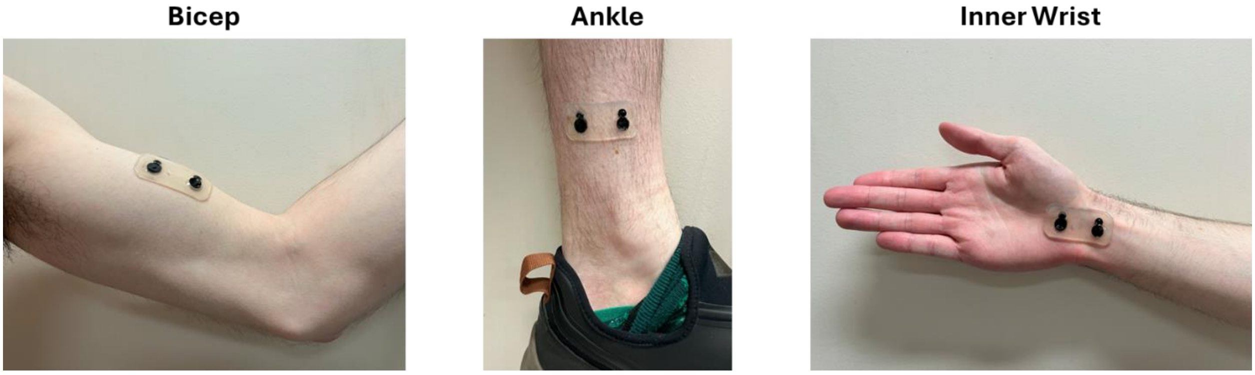

Figure S2. Hydrogel-based sensors placed on various parts of the body.

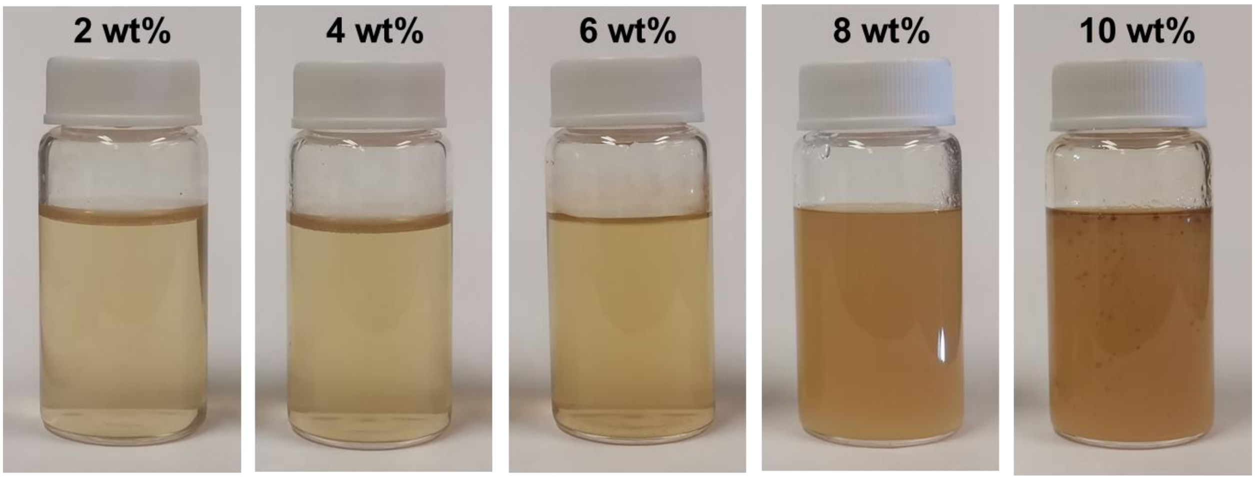

Figure S3. Photographs showing pyrrole aqueous solutions with different pyrrole contents from 2 wt% to 10 wt%.

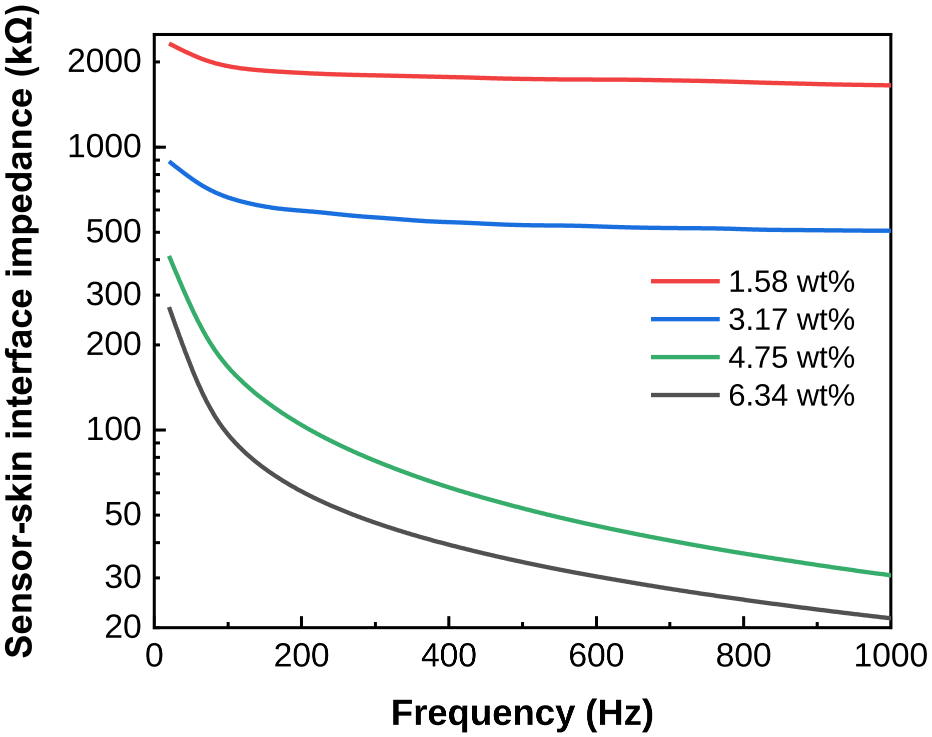

Figure S4. Sensor-skin interface impedance of hydrogel-based sensors with different polypyrrole contents.

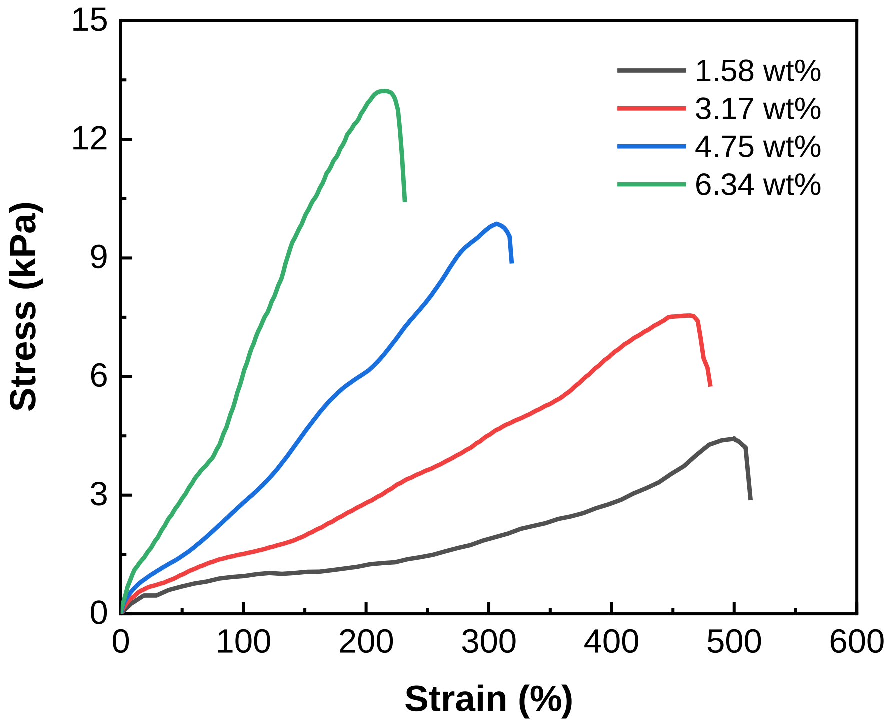

Figure S5. Strain-stress curves of hydrogels with different polypyrrole contents.

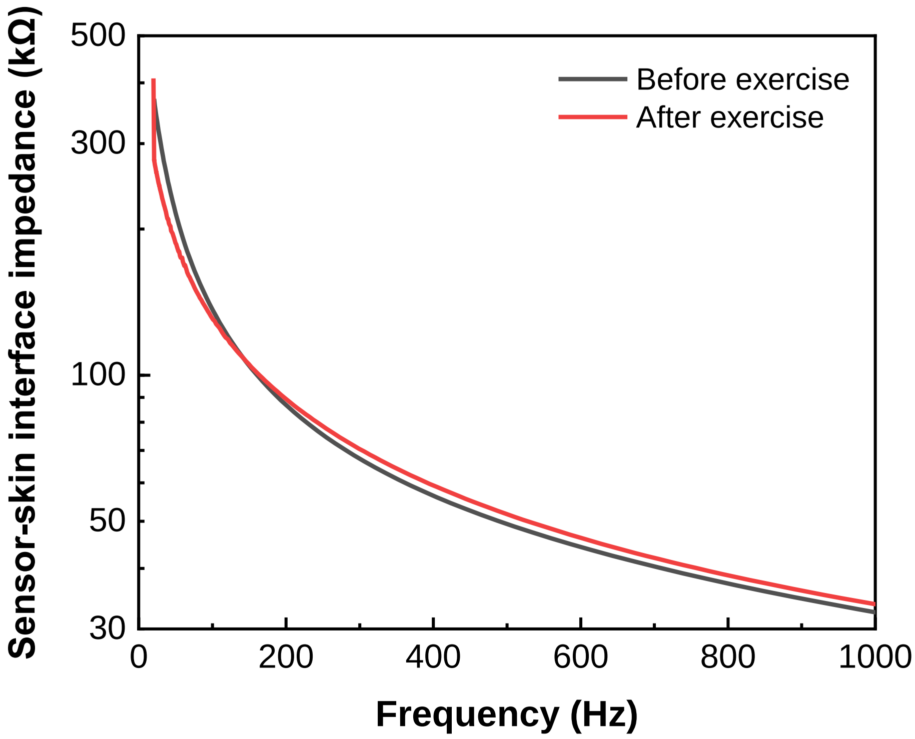

Figure S6. Sensor-skin interface impedance of hydrogel-based sensors before and after exercise within the frequency range of 20-1000 Hz.

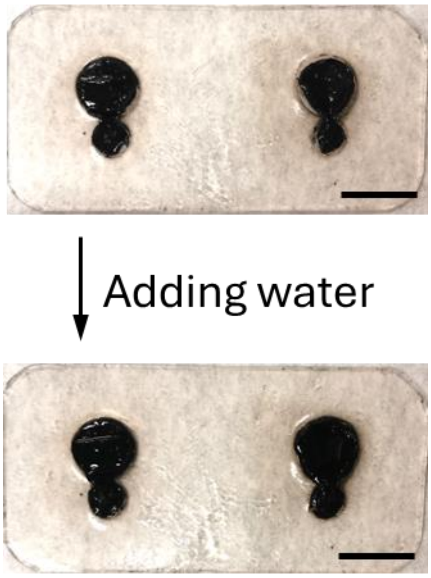

Figure S7. The photographs showing a sensor after being stored for 2 months in the ambient environment and 15 minutes after adding a drop of water to each electrode in the sensor to rehydrate it. Scale bar is 1 cm.
